# Effect of potato flour on quality and staling properties of wheat–potato flour bread

**DOI:** 10.1002/fsn3.1829

**Published:** 2020-08-31

**Authors:** Qian Ju, Yaoxi Li, Huaxing Sun, Jincheng Chen, Yanqiu Yuan, Yayun Hu, Kaori Fujita, Guangzhong Luan

**Affiliations:** ^1^ College of Food Science and Engineering Northwest A&F University Yangling China; ^2^ Japan International Research Center for Agricultural Science Ibaraki Japan

**Keywords:** potato flour, staling, texture property, water migration, X‐ray diffraction

## Abstract

To elucidate the impact of potato flour (PF) on quality changes and staling characteristics of the composite bread from wheat–potato flour (WPF), the physicochemical (specific volume, colority, sensory value, texture, and viscosity) properties, and staling (X‐ray diffraction and water migration) properties of bread were investigated. The quality of composite bread was comparable to wheat bread when addition level of PF at 20%, but decreased when the addition level increased to 30% or more, and became unacceptable at 50%. A chewy mouthfeel and an elastic and none‐crumbly texture were observed on composite bread, which had higher hardness than wheat bread, and could keep on both longer linear distance and higher linear force during compression test. It indicated that such new parameters other than hardness should be introduced to coordinate with the texture quality of composite bread. During storage, the higher addition level of PF significantly decreased crystallinity of composite bread and slowed water migration rate from the crumb to crust, suggesting that PF had antistaling effect on composite bread, which was further emphasized by the fact that the setback value of the WPF decreased with the increase of PF addition.

## Graphical abstract

This study evaluated the quality and staling properties of composite bread, which formulated with different levels (0%, 20%, 30%, 40%, 50%) of potato flour. From the results, we found that the quality of composite bread was comparable to wheat bread when addition level of PF at 20%, and the addition of potato flour had an anti‐staling effect on composite bread.

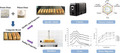

## 1 INTRODUCTION

Potato (*Solanum tuberosum* L.) is a valuable tuber crop planted worldwide (Friedman, 2006), and an important material in food industry. In 2016–2017 crop year, 388 Mt of potatoes were produced in the world, and China was the largest producer with a production of 194 Mt (Zhao et al., 2020). China launched the national strategy “Staplization (means the application of an ingredient as staple food) of potato” in 2016 for the reason of food security, pushing the application of potato into bread, noodles, crackers, and other staple foods (Pang, Qu, & Guo, 2018). Besides starch, potato flour (PF) is another important industrial product, which contains all the dry matter except the potato peel (Zhao, Wang, & He, 2018). The processing of PF is simpler than starch and could improve the fermentation of dough, giving bread various colors, unique flavor, taste, and nutrition as well (Jemziya & Mahendran, 2017). Introduction of PF to wheat foods could improve the comprehensive utilization efficiency of potato and expand its application. Furthermore, it could decrease the intake of gluten content for special needs, thus reduces the risk of celiac disease (CD) (Joshi, Sagar, Sharma, & Singh, 2018).

Bread staling results in loss of flavor and texture, and mainly leads to the increase of crumb firmness and loss of freshness, as well as severe waste (Fadda, Sanguinetti, Del Caro, Collar, & Piga, 2014). There are three main theories on the mechanism of bread aging: first, the transfer of the moisture in bread; second, the recrystallization of starch; and third, the interaction between starch and gluten in bread (Zhan, Ren, Min, & Liu, 2013). To restrict the bread staling caused by starch retrogradation, pregelatinized wheat, and maize starches could be used as antistaling additives (Hesso, Loisel, Chevallier, & Le‐Bail, 2014). Moreover, bread with extruded potato starch was observed at a lower value of retrogradation rate (Gumul, Krystyjan, Buksa, Ziobro, & ZieBa, 2014). The addition of potato flour to bakery products slowed down the staling rate as a fresh‐keeping agent (Joshi et al., 2018). However, more information is needed to evidence the antistaling efficiency of potato flour and look inside the mechanism. Consequently, the objectives of the present study were to assess the impact of PF on the quality of the composite bread and to elucidate the staling mechanism explanation of composite bread.

## 2 MATERIALS AND METHODS

### 2.1 Materials

The high‐gluten wheat flour (WF, 10.75% moisture, 55.58% starch, 12.80% protein, 0.48% ash) was provided by Dacheng Food Ltd. Commercial potato flake (Shandong University of Technology) was milled using a universal high‐speed smashing machine (FW‐100D, Tianjin Xinbode Instrument Co., Ltd.) and passed a sieve of 200 mesh. Then, the potato flour (PF, 6.62% moisture, 59.26% starch, 9.16% protein, 0.29% ash, and 90.72% degree of gelatinization, which were determined under the method of Xiong (2000) for the degree of gelatinization, AACC Methods (2000) for moisture, protein, and ash content, AACC Method 76‐11 (1999) for starch content) was packed in a resealable polyethylene bag for further study.

### 2.2 Methods

#### 2.2.1 Bread preparation

Bread preparation was performed according to the method of GB/T 14,611—2008. The formula (flour weight‐based) of wheat bread (WB) and bread from WPF (WPFB) with substitutive levels at 20%, 30%, 40%, and 50% (WPFB20, WPFB30, WPFB40, and WPFB50) contained the following ingredients: WF or WPF, 2% instant dry yeast, 1% salt, 8% sugar, 5% nonfat dry milk, and 4% shortening. According to pre‐elaborations, the amount of water for each formula was calculated by the sum of the 60% weight of WF and 120% of PF. A straight‐dough method was performed with a five‐speed dough mixer (SM‐1688, Shepherd Wang Electrical Hardware Co., Ltd). Dry ingredients (WPF, instant dry yeast, and nonfat dry milk) were blended for 2 min at speed 1. Then, water and solution of sugar and salt were added to develop a dough by mixing for 1 min at speed 1, 1 min at speed 2, 1 min at speed 3 after the shortening was added, and 2 min at speed 5.

The resultant doughs were kneaded and rounded manually. After fermented for 90 min in a proofer (HSW‐400, Shanghai Jinghong Experimental Equipment Co., Ltd.) at 30°C and RH of 85%, doughs were sheeted with the noodle pressing machine (300/100 type, Hubei WuRui Machinery Equipment Co., Ltd.) for three times in order to be bubble‐expelled, and then rolled up manually and placed into baking tins (15 × 6 × 6.5 cm) for wakeup‐proof for 20 min at 38℃ and RH of 85%. Bread billets were baked in the oven for 20 min at top temperature of 180℃ and bottom 190℃. After baking, the loaves were cooled for 1 hr at room temperature and stored in resealable polyethylene bags for further analysis. The bread preparation for each formula was performed in triplicate.

#### 2.2.2 Evaluation of the physical characteristics of bread

##### The specific volume

After cooling for 2 hr, the weight and volume of bread were measured. Bread loaf volume was determined using the rapeseed displacement method of AACC International (2001). The specific volume was calculated by dividing the volume by the loaf weight (expressed as the loaf volume of 100 g of bread, i.e., cm^3^/g).

##### The color

The color of crust and crumb was measured using the colorimeter (X‐rite color technology Co., Ltd., Ci7600, America) at an angle of 10° based on the CIE L*, a*, b* system. The C* value, which indicated the color saturation, and calculated by $\sqrt{{\left(\text{a*}\right)}^{2}+{\left(\text{b*}\right)}^{2}}$ was used to evaluated the colority as well. Crumb and crust color was determined at four different points on each piece of bread (Sun et al., 2019), and each measurement was performed in triplicate.

##### The mechanical test

The mechanical test was conducted by using a TA. XT Plus texture analyzer (Stable Micro systems Ltd). The cubical pieces (2 × 2×2 cm) were cut from the central part of crumbs and compressed using a p/36 probe to a strain of 40% at the rate of 1 mm/s. Each measurement was done in five to eight replications after storage for 2, 24, and 48 hr, respectively, after baking, according to the method of Ji et al. (2017). The hardness was defined as the maximum force during the compression.

**FIGURE 1:**
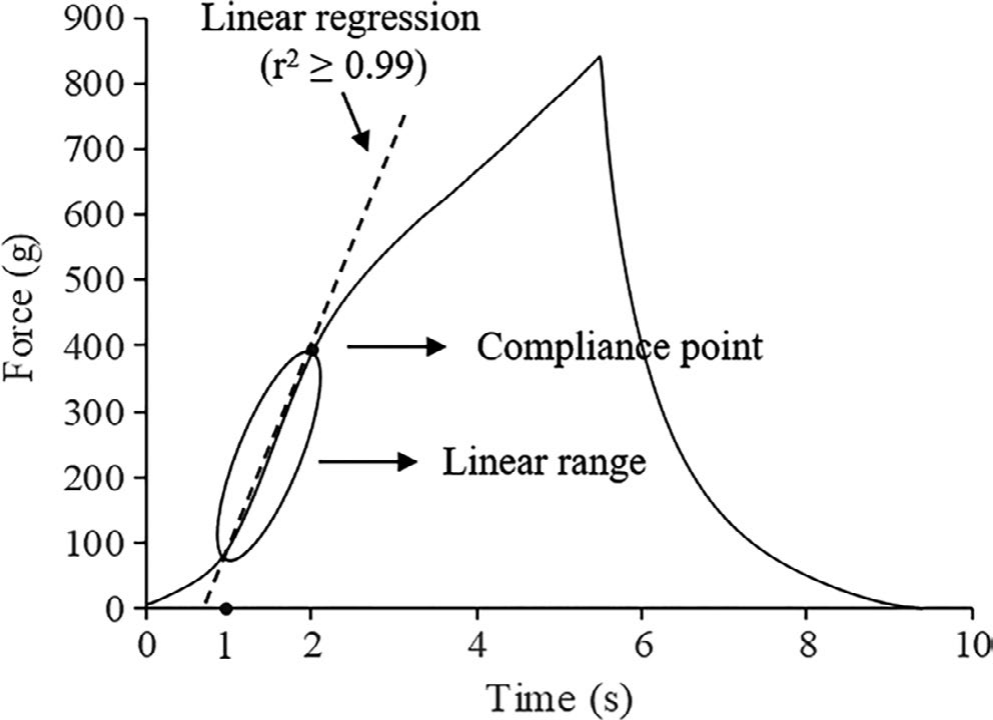
The diagram for selecting linear range from compression curve

The linear range was conducted by selecting the starting point at 1 s, and the ending point which the determinant coefficients of the linear regression equation was not <.99 (*r* ^2^ ≥ .99). The linear distance and linear force were defined as the abscissa and ordinate of the ending point respectively, whereas the chord modulus of elasticity was defined as the slope of the linear regression line (Figure 1).

#### 2.2.3 Sensory evaluation

The methodology of the test was reviewed and approved by Northwest A&F University and informed consent was obtained from each subject prior to their participation.

The 9‐point hedonic scale (9 = like extremely, 5 = neither like, nor dislike, 1 = dislike extremely) was performed for the hedonic expression of data, which consist of panelists (three males and four females) who were experienced in descriptive sensory studies (Kim, Yun, & Jeong, 2015). The sensory attributes including color, cell uniformity, odor, taste, texture, and overall acceptability were defined by relevant literature (Jeddoua et al., 2017). For the sensory analysis, each bread sample was cut into slice (1 × 1 × 2 cm) and placed in a white plastic pan randomly encoded by three numbers.

#### 2.2.4 Observation of Water immigration during bread storage

Bread samples were packed into polyethylene resealable bags after cooling for 1 hr, and stored at 20 ± 2℃, RH of 35% for 1, 3, 5, and 7 days for further analysis. The bread being cooled for 1 hr after baking was used as the sample for 0 day.

The moisture of both the crust and crumb from above samples were determined by drying 2 g of the samples in an oven (DHG‐9140A, Shanghai Jinghong Experimental Equipment Co., Ltd.) at 103°C for 24 hr, following AACC Methods 44‐15A (2000).

#### 2.2.5 X‐ray diffraction (XRD) analysis

The characteristics of X‐ray diffraction for samples described in 2.2.4 (stored at 20 ± 2°C, RH of 35% for 1, 3, 5, and 7 days) was freeze‐dried (GENESIS Freeze Dryer, IRTIS25XL), and samples of ground crumbs were scanned using a Bruker D8 Advanced Diffractometer (BRUKER Corp.) at 40 kV, 35 mA. The scanning region of the diffraction angle(2θ) was 4–50°, and the step size was 0.02 (Aguirre et al., 2011).

The patterns of XRD were fitted using Origin 7.0. Crystalline peaks and amorphous areas were quantified with MDI Jade version 5.0 software. Each diffraction was performed in several times until the result was stable. The degree of crystallinity was described as total mass crystallinity (TC) which was calculated by formula 1 (Demirkesen, Campanella, Sumnu, Sahin, & Hamaker, 2014).

TC = Ic/(Ic + Ia) (Formula 1).

where Ic is the integrated intensity of the crystalline phase, and Ia is the integrated intensity of the amorphous phase.

#### 2.2.6 Pasting properties of flours

Pasting behaviors of the mixture of WF, PF, and premixed flour of WF and PF (WPF) with substitutive level at 20%, 30%, 40%, and 50%, respectively, were analyzed with a Rapid Visco‐Analyzer (RVA, S/N 2153539‐TMB, Perten, Australia), according to the method of LS/T 6101–2002. The results form RVA include peak viscosity (the maximum viscosity during pasting), trough (the minimum viscosity during cooldown after the sample reached to peak viscosity), breakdown (the difference between the peak viscosity and the minimum viscosity during pasting), final viscosity (the viscosity at the end of RVA test), setback (the difference between final viscosity and trough), peak time (the time to reach the peak viscosity), and peak temperature (the temperature at which viscosity begins to increase).

#### 2.2.7 Statistical analysis

All the data were submitted to the one‐way analysis of variance (ANOVA) and Turkey's test (*p* ≤ .05) which was used to describe means with 95% confidence intervals. Statistical analyses were performed using DPS software version 7.0.

## 3 RESULTS AND DISCUSSION

### 3.1 Effect of potato flour on the physical properties of breads

With the increasing of PF addition, the L* value of WPFB was increased, both a* and b* values were decreased (Table 1). Moreover, the significant (*p* < .05) changes of C* value explaining lighter colors of crust compared to control. PF addition resulted in both lower loaf volume and height, leaving the crust a further distance to the top heating elements of the oven during baking. Consequently, inadequate Maillard reaction during baking resulted in the pale crust and loss of the yellow‐brown color which is characterized in the baking product (Joshi et al., 2018).

**TABLE 1:** Color values and specific volume of bread samples prepared with different potato flour addition

Samples	Crust color				Crumb color				Specific volume
	L*	a*	b*	C*	L*	a*	b*	C*	
WFB	56.47 ± 0.32^b^	16.56 ± 0.18^a^	25.57 ± 0.41^a^	30.36 ± 0.45^a^	81.53 ± 1.02^a^	−0.28 ± 0.08^bc^	13.51 ± 1.37^b^	13.51 ± 1.37^c^	2.76 ± 0.12^a^
WPFB20	56.80 ± 0.25^b^	16.50 ± 0.31^a^	25.66 ± 1.31^a^	30.50 ± 1.35^a^	79.38 ± 0.68^ab^	−0.21 ± 0.04^ab^	16.50 ± 0.16^a^	16.50 ± 0.16^b^	2.12 ± 0.12^b^
WPFB30	66.71 ± 1.26^a^	10.35 ± 1.30^b^	25.21 ± 0.61^a^	27.25 ± 1.44^b^	78.68 ± 0.97^b^	−0.17 ± 0.01^ab^	16.63 ± 0.65^a^	16.63 ± 0.65^b^	1.82 ± 0.07^bc^
WPFB40	71.72 ± 1.83^a^	4.11 ± 1.52^c^	24.4 ± 2.27^a^	24.74 ± 2.73^b^	75.47 ± 1.19^c^	−0.11 ± 0.02^a^	17.67 ± 0.58^a^	17.67 ± 0.58^a^	1.58 ± 0.16^c^
WPFB50	74.02 ± 1.09^a^	1.67 ± 0.55^d^	21.73 ± 0.40^b^	21.79 ± 0.68^c^	75.25 ± 1.08^c^	−0.34 ± 0.09^c^	17.98 ± 0.65^a^	17.98 ± 0.66^a^	1.41 ± 0.08^c^

Note

WFB: wheat flour bread; WPFB20, WPFB30, WPFB40, WPFB50: wheat–potato flour bread with 20%, 30%, 40%, 50% potato flour addition respectively. L*: luminosity. a*: red index. b*: yellow index. Different superscript letters at the same column indicate significantly different(*p < *.05).

In the case of crumb, the L* value decreased from 81.53 to 75.25, whereas the b* value increased from 13.51 to 17.98, which demonstrated the bread from WPFB toward yellow. The cooking process during PF production would affect the pigment content and darken the flour color (Martínez, Oliete, & Gómez, 2013).

With the increase of PF addition, the specific volume of bread decreased from 2.76 to 1.41 (Table 1). During leavening, the carbon dioxide could not be retained in the dough effectively due to PF addition, which diluted the gluten content and reduced the dough gas‐holding capacity. Therefore, an inadequate expanded gluten network in dough resulted in a decreasing of bread specific volume (Pongjaruvat, Methacanon, Seetapan, Fuongfuchat, & Gamonpilas, 2014).

### 3.2 Sensory analysis of composite breads

**FIGURE 2:**
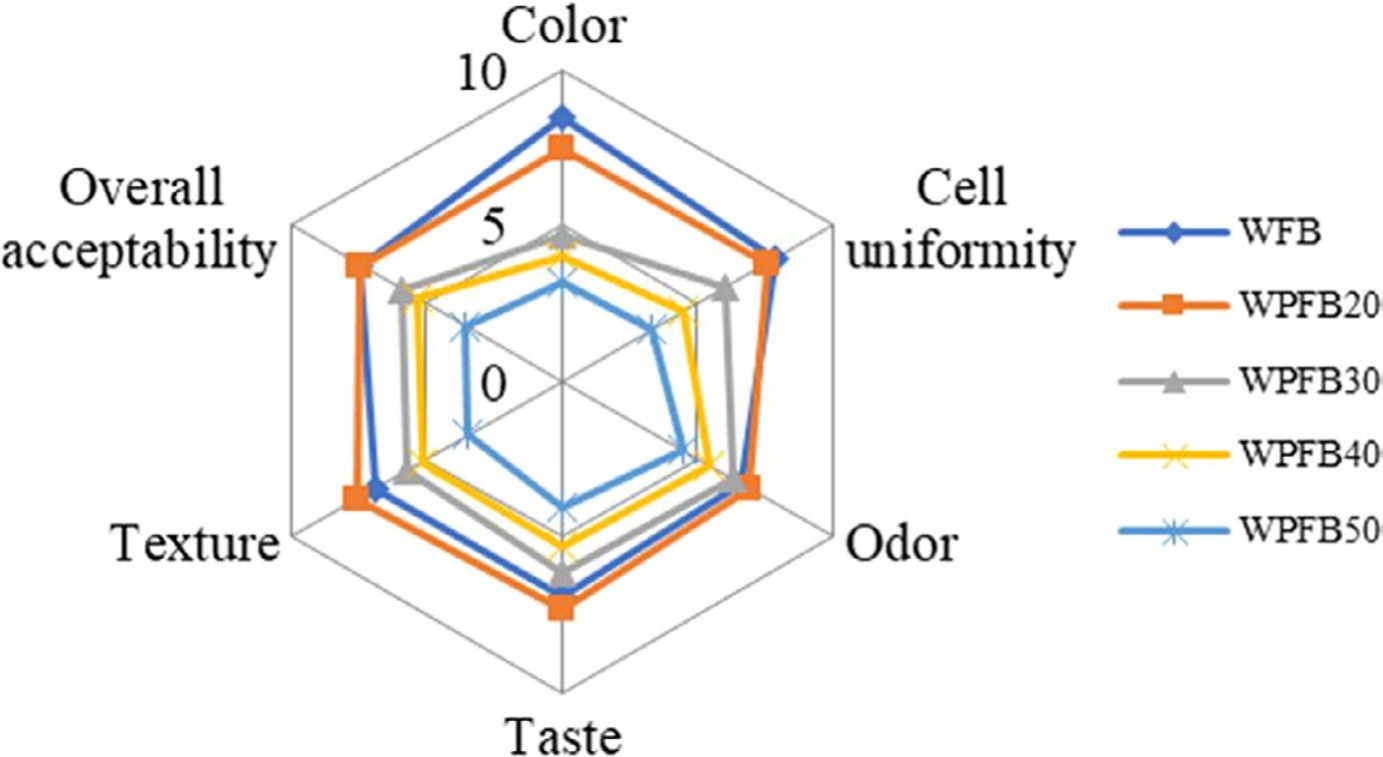
Spider plot of the bread sensory evaluation prepared with different addition potato flour. WFB: wheat flour bread; WPFB20, WPFB30, WPFB40, and WPFB50: wheat–potato flour bread with 20%, 30%, 40%, and 50% potato flour addition, respectively

The spider plot (Figure 2) demonstrated the organoleptic quality analysis of bread samples. For overall acceptable, the scores of WPFB20 were comparable with WFB, achieving the highest score of 7.43, and all WPFB samples were acceptable since the scores were higher than 5, except WPFB50 with a score of 3.57. WPFB20 obtained the highest score for all sensory characteristics except the color and cell uniformity. Similar dependence was studied by Kim et al. (2015), which illustrated that rice bread was fortified with 20% potato starch showed the highest scores for overall acceptability. But when the substitutional level increased to 30% or more, the quality of the WPFB decreased in color, texture, and taste, and became unacceptable at the addition level of 50%.

### 3.3 Water content of crumb and crust during storage

**FIGURE 3:**
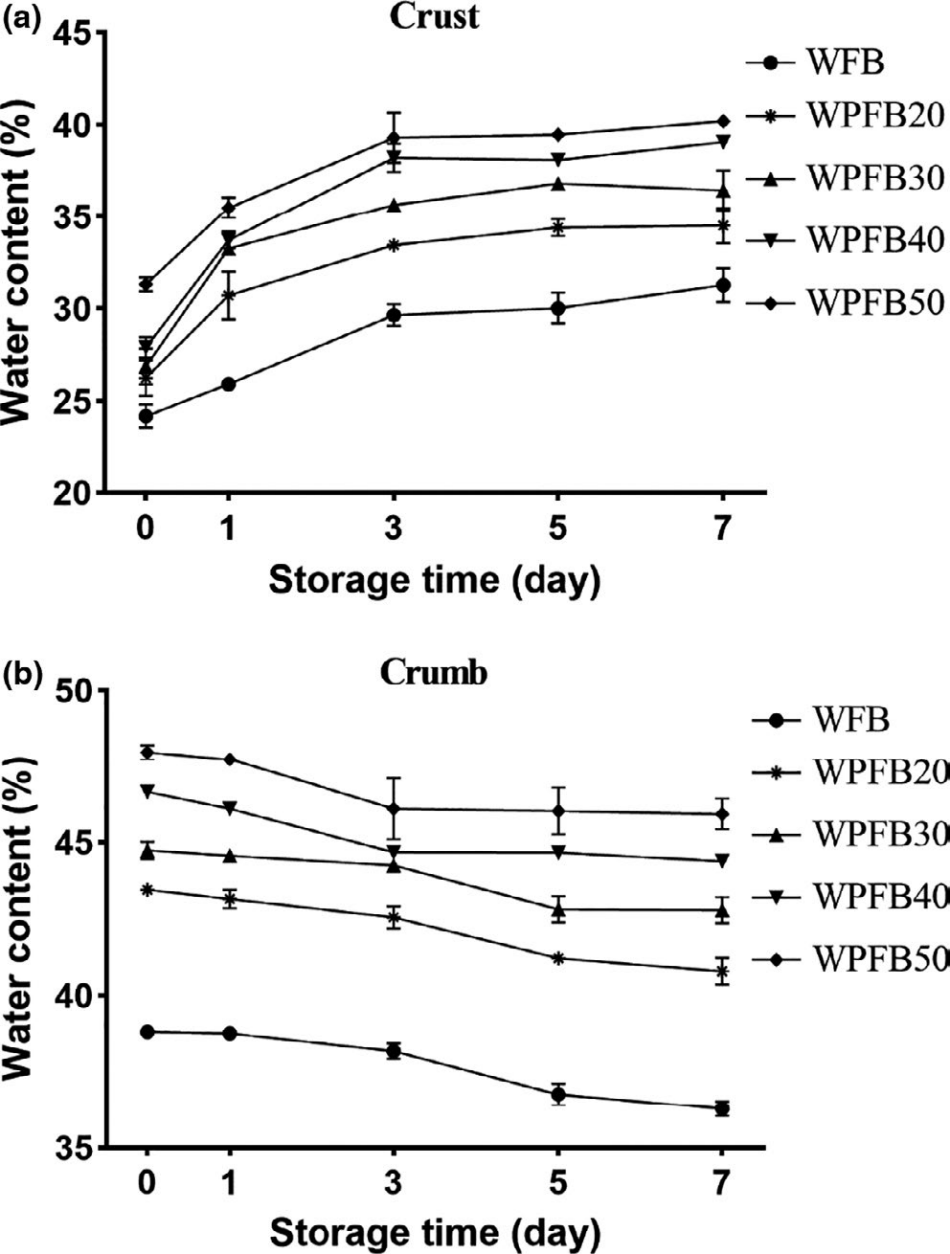
Water content of wheat and PF bread crust (a) and crumb (b) during 7 days storage times. WFB: wheat flour bread; WPFB20, WPFB30, WPFB40, WPFB50: wheat–potato flour bread with 20%, 30%, 40%, and 50% potato flour addition, respectively

Figure 3a,b described the water content of crust and crumb for WFB and WPFBs at different storage time. There were two stages for water changes of crumb and crust: a sharp increase or decline in first three days storage and, a slowly change during the late period of storage, respectively. This was also consistent with the crystallinity changes mentioned later. This is due to the moisture exchange between the crust and crumb at the early storage period. After three days, the moisture in the bread system reached an equilibrium gradually. Therefore, in the later storage period, the moisture exchange has slowed down. After storage for 7 days, the water content of the WFB crumb decreased from 38.82% to 36.3%, while WPFB50, changed from 47.97% to 45.93%; on the contrary, the water content of WFB crust raised from 24.2% in fresh to 31.29%, while WPFB50 was from 31.33% to 40.2%. Water migration from crumb to crust during bread staling was a result of the higher water activity of crumb (Ronda, Caballero, Quilez, & Roos, 2011). Moreover, water played an important role in crumb firmness due to its plasticizing effect on the crumb network, the loss of water content would lead to the hardening of bread crumb, worsening taste and faster aging (Moo‐Yeol & Chinachoti, 2000).

Compared with WFB, the lines of WPFB showed flatter especially after 3 days storage time, inferred the slower moisture migration and diffusion. For both crust (Figure 3a) and crumb (Figure 3b), all WPFBs had a higher water content than WFB during 7 days storage. The starch in PF was in the gelatinized state, which had a high water‐binding ability, so the PF could be used as a humectant to prevent moisture loss during bread aging (Joshi et al., 2018). On the other hand, high water content of bread could decrease hardening rate (He & Hoseney, 1990).

### 3.4 Effect of potato flour on texture properties during bread storage

**FIGURE 4:**
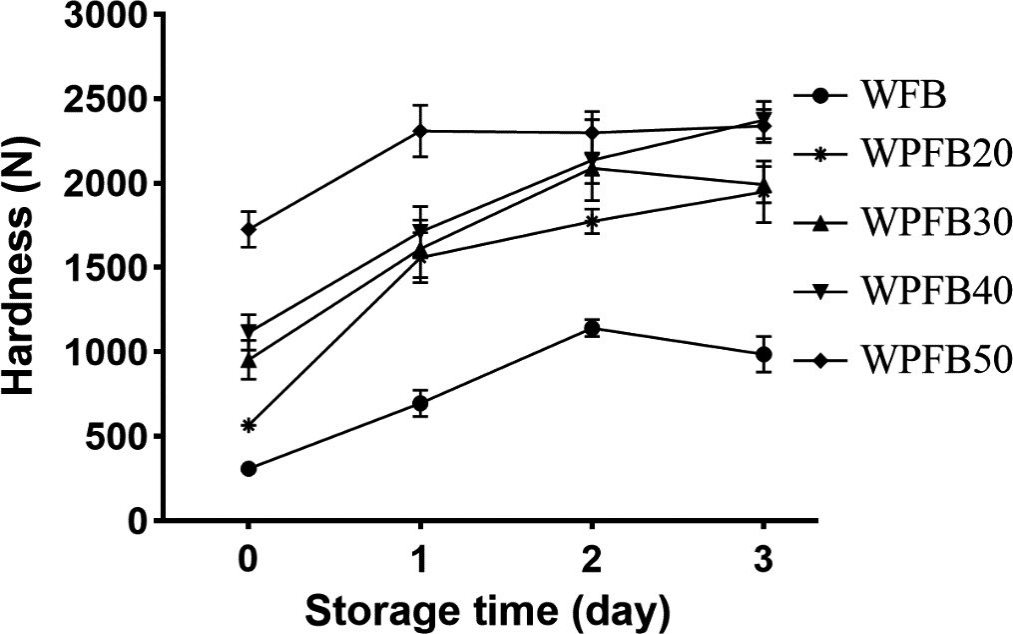
Hardness changes of wheat and composite breads during storage. WFB: wheat flour bread; WPFB20, WPFB30, WPFB40, and WPFB50: wheat–potato flour bread with 20%, 30%, 40%, and 50% potato flour addition, respectively

The hardness of the WPFB crumb (Figure 4) increased significantly with PF addition (*p* < .05). It may due to the low specific volume of WPFB, which gave bread a firmer crumb, leading to the high bread hardness. Moisture change would accelerate the starch–gluten and starch–starch interaction thus hardening the bread crumb (Ozkoc, Sumnu, Sahin, & Turabi, 2009). Because of the stronger water absorption of PF, the formula of WPFB contained higher water content, which led to the full swelling and dissolving of starch, promoting the contact of the surface between starch and gluten, and thus increased the hardness of bread crumb (Gumul et al., 2014).

As mentioned in 3.6, during bread staling, the crystallinity of stale bread declined with PF addition, whereas the hardness increased. Moreover, during the experiment we have found that the texture of the fresh and stale bread crumb of WPFB were chewy and elastic, rather than crumbly compared to WFB.

**FIGURE 5:**
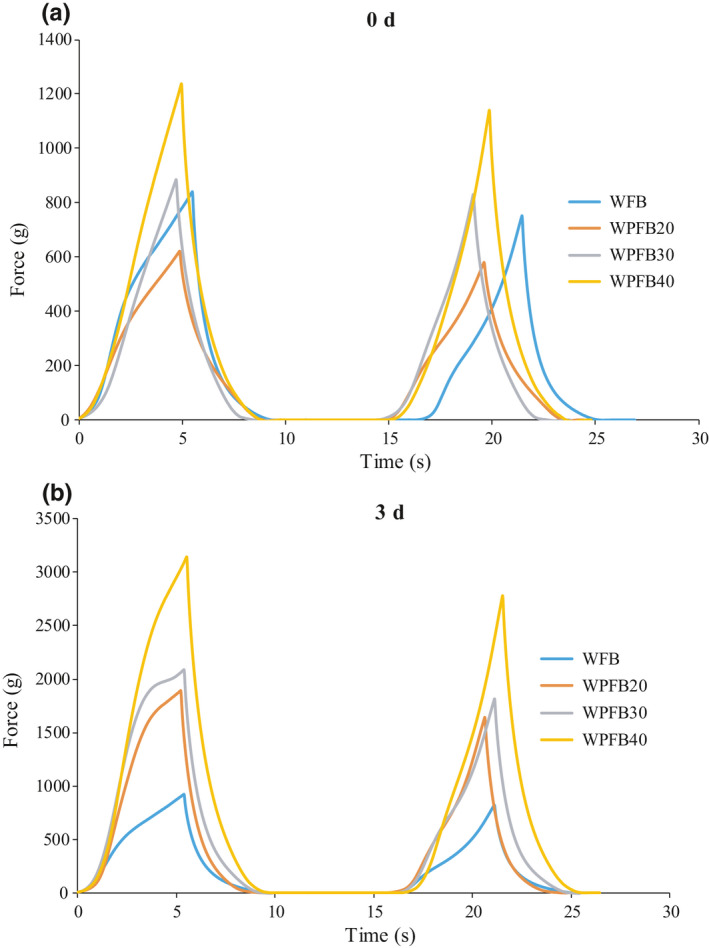
(a) The compression curve of the fresh bread samples. (b) The compression curve of the stored bread samples for 3 days

Based on the interesting finding, we further analyzed the compression curve of the bread samples (Figure 5). The linear distance, linear force, and chord modulus of elasticity, i.e. the slope of linear range curve were shown in Table 2. For both fresh and staled bread samples, which contained more PF had both longer linear distance and higher linear force at the same time. It was inferred that the addition of potato flour could prevent bread crumb from slagging, and give bread a chewy mouthfeel.

**TABLE 2:** The parameters’ values derived from the linear range of compression curve from different bread samples

Samples	Storage times	Linear parameters		
		Linear distance (mm)	Linear force (g)	Elasticity (g/mm)
WFB	0 day	2.02	392.2	309.3
	3 days	1.93	422	307.05
WPFB20	0 days	2.16	324.1	184.29
	3 days	2.7	1,178.1	700.19
WPFB30	0d ay	4.705	884.6	235.78
	3 days	2.775	1531.6	839.6
WPFB40	0 day	4.965	1,237.1	298.29
	3days	3.12	1998.8	1,025.8

### 3.5 X‐ray diffraction

**FIGURE 6:**
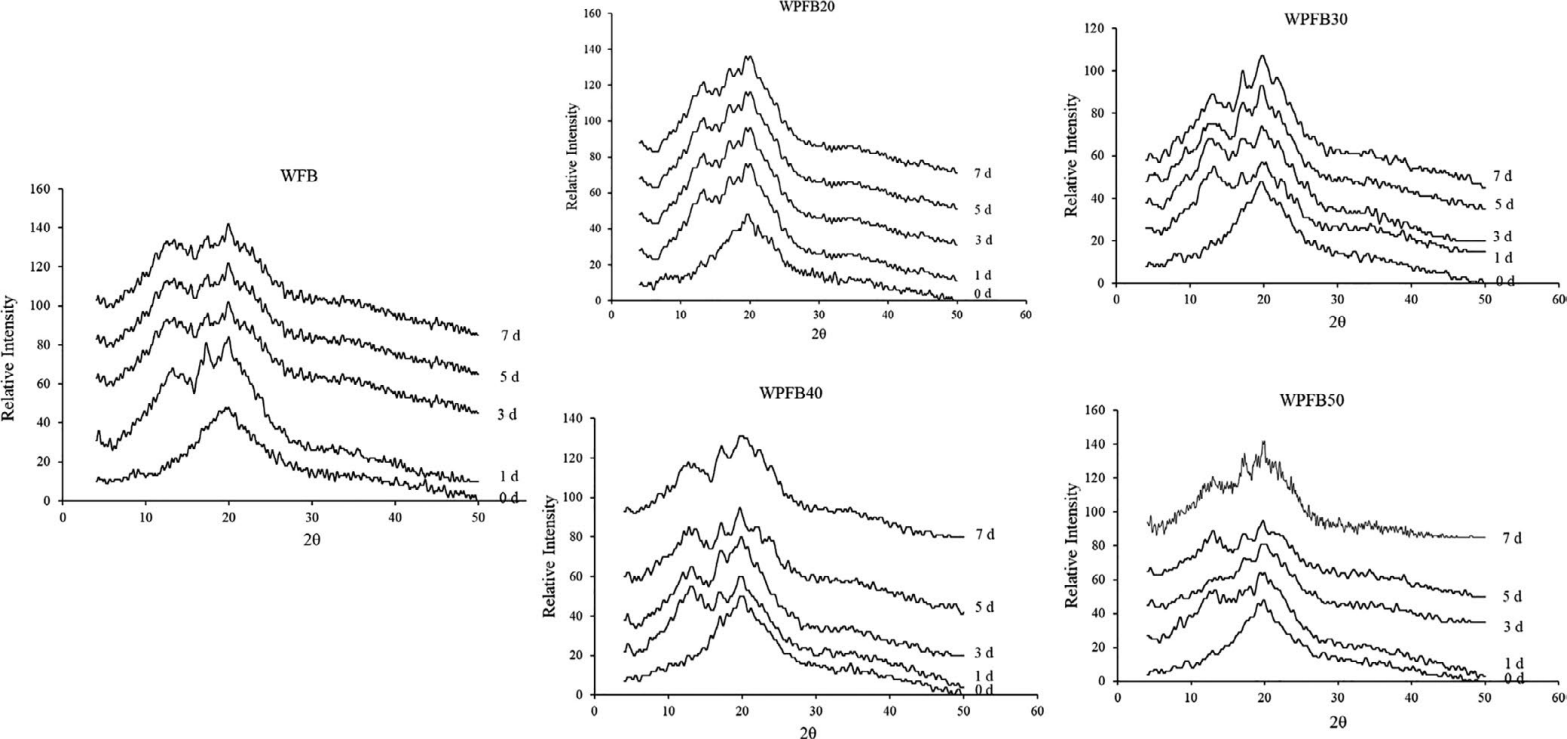
XRD patterns of bread crumb after stored for different times (0, 1, 3, 5, and 7 days). WFB: wheat flour bread; WPFB20, WPFB30, WPFB40, and WPFB50: wheat–potato flour bread with 20%, 30%, 40%, and 50% potato flour addition, respectively

Figure 6a–e showed the X‐ray diffraction diagrams of fresh (after baking 1 hr) and stored (1, 3, 5, and 7 days) bread crumbs which containing different amounts of PF. For all patterns of fresh bread, only one peak can be observed around 19.5 ~ 20°, corresponding to the V‐type structure, it indicated helical amylose complexes formed by amylose complexing and fatty acids. These results were consistent to the studies by Aguirre et al. (2011) and Demirkesen et al. (2014). During storage, all bread samples appeared a pattern of B‐type with the diffraction peaks at 13.2°, 17.2°, and 20°, which superposed a V‐type structure indicated the peak at 20° and a weak A‐type structure. B‐type crystals were produced during staling as the recrystallization of gelatinized starch and water transfer from the amorphous phase to the crystalline phase (Demirkesen et al., 2014; Kang, Reddy, Park, Choi, & Lim, 2018). Moreover, the B‐type crystal also reflected the role of potato flours which was the tuberous starch.

**FIGURE 7:**
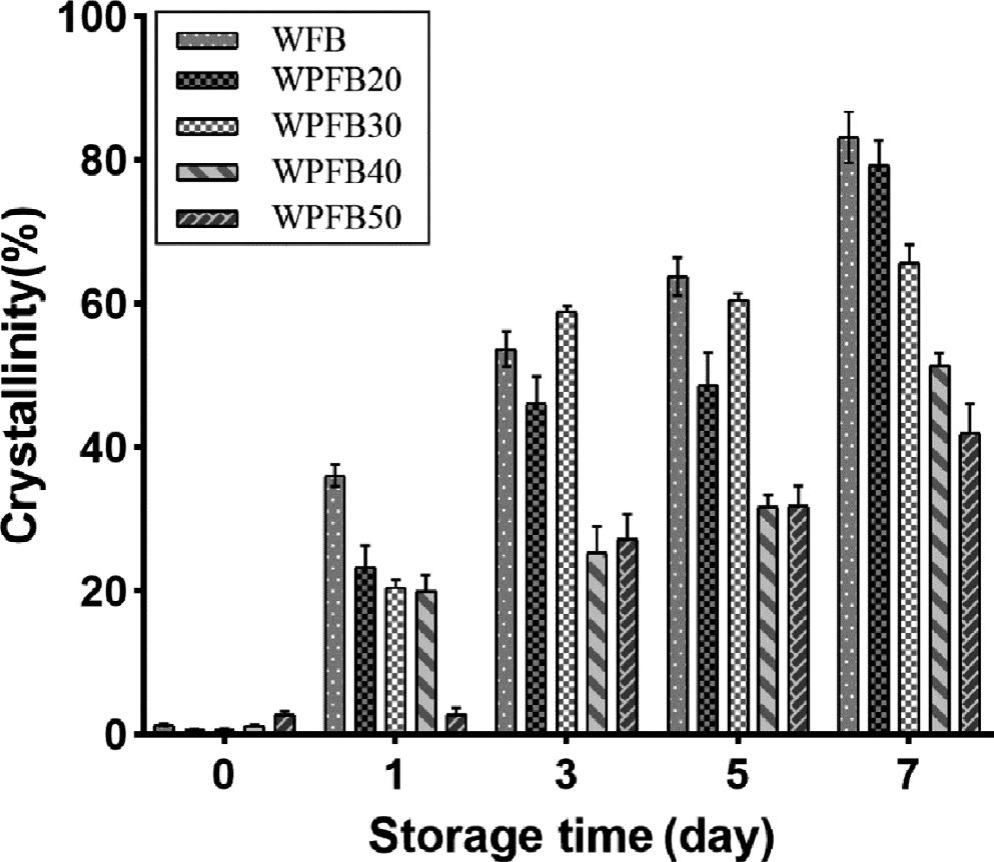
Total mass crystallinity grade (TC) of different storage time bread. WFB: wheat flour bread; WPFB20, WPFB30, WPFB40, and WPFB50: wheat–potato flour bread with 20%, 30%, 40%, and 50% potato flour addition, respectively

Crystallinity grade analysis was used to characterize the aging degree of stored bread (Song & Tong, 2017). As shown in Figure 7, with storage time went on, the total mass crystallinity (TC) of all bread samples was significantly increased (*p* ≤ .05). The crystallization grade increased rapidly in the former 24 hr and then slowed down (Figure 7). It was mainly caused by the retrogradation of amylose occurred in a few minutes to the first few days of storaging at a faster rate, and the amylopectin was gradually regenerated at a slow rate in the late storage (Sullivan, Hughes, Cockman, & Small, 2017). The sample with the highest degree of crystallinity was found to be the WFB after stored for 7 days. In the same storage time, with the increase of the PF addition, the crystallinity decreased significantly, which strongly indicated that the addition of PF could inhibit the staling of bread. This was probably because the PF broke the gluten network in the system, hindered the process of water diffusion and transfer, as well as the interaction of starch and protein (Joshi et al., 2018). This point was strengthened by the decreasing of setback values mentioned in 3.6 section (Table 3).

**TABLE 3:** Pasting indexes of WF, PF, and WPF

Samples	Peak Visco./cp	Trough/cp	Breakdown/cp	Final Visco./cp	Setback/cp	Peak Time/min	Pasting Temperature/℃
WF	1635.00 ± 48.79^ab^	979.00 ± 35.36^ab^	656.00 ± 13.44^b^	2034.00 ± 48.79^a^	1,055.00 ± 13.44^a^	5.93 ± 0.00^a^	95.00 ± 0.00^a^
PF	831.00 ± 52.33^d^	529.00 ± 53.74^d^	302.00 ± 16.07^d^	797.00 ± 74.95^d^	268.00 ± 21.21^d^	1.20 ± 0.52^c^	51.05 ± 0.00^c^
WPF20	1,820.50 ± 10.61^a^	1,031.50 ± 12.02^a^	789.00 ± 1.41^a^	1921.50 ± 2.12^a^	890.00 ± 9.90^b^	5.80 ± 0.00^a^	70.20 ± 0.07^b^
WPF30	1557.00 ± 8.49^b^	901.00 ± 8.49^b^	656.00 ± 16.97^b^	1678.00 ± 2.83^b^	777.00 ± 5.66^bc^	5.73 ± 0.00^ab^	70.23 ± 0.04^b^
WPF40	1,449.59 ± 9.70^bc^	952.00 ± 18.59^ab^	497.50 ± 38.89^c^	1507.00 ± 32.53^b^	555.00 ± 106.07^c^	5.47 ± 0.00^b^	69.35 ± 0.07^b^
WPF50	1,257.00 ± 96.17^c^	774.00 ± 57.98^c^	483.00 ± 38.18^c^	1,340.50 ± 102.5^c^	566.50 ± 44.55^c^	5.40 ± 0.00^b^	69.70 ± 0.57^b^

Note

WF: wheat flour; PF, potato flour; WPF20, WPF30, WPF40, and WPF50: premixed flours of WF and PF with potato flour substitutive level at 20%, 30%, 40%, and 50%, respectively. Different superscript letters at the same column indicate significantly different(*p <* .05).

### 3.6 Pasting characteristics of flours

The indexes of viscosity evolving of WF, PF, and WPF during the programmed pasting process were shown in Table 3. Peak viscosity suggested the ability of water‐binding, reflecting the strength of stickiness (Bhattacharya, 2012; Yan, Gao, Xing, & Zhang, 2016). With an increasing addition of PF from the level of 20% to 50%, there was a decrease from 1,820.5 to 1,257 presenting on the peak viscosity of WPF, while the peak viscosity value of WF was 1635. Similarly, with the increasing of PF substitution, the through, breakdown, and final viscosity values had suffered different levels of decrease. These results demonstrated that PF had a great influence on the pasting viscosity of the mixture flours system. The potato fiber and protein in the mixture flours could prevent the formation of network within starch structure. Meanwhile, the nonstarch polysaccharides also compete to combine water, which obstructed the swelling of the starch, and thus reduced the gelatinized viscosity value (Guo, Li, & Zhang, 2015). The cooking process of PF could cause the starch to lose ordered structure and decrease paste viscosity (Martínez et al., 2013). The setback value of the WPF decreased with the increase of PF addition, indicating better stability of cold paste, slower retrogradation rate, and antistaling potential of PF (Yan et al., 2016).

### 3.7 Correlation analysis

The hardness showed a significant linear correlation with the crumb moisture and crystallinity, except for WPB 50. Among them, the hardness was negatively correlated with crumb moisture (with the correlation coefficient were −0.859, −0.906, −0.951, and −0.977 corresponding to WFB, WPB20, WPB30, and WPB40), and positively correlated with crystallinity (with the correlation coefficient were 0.995, 0.969, 0.945, and 0.942 corresponding to WFB, WPB20, WPB30, and WPB40). There was a significant negative correlation between the crystallinity and crumb moisture, with the correlation coefficient were −0.872, −0.912, −0.807, −0.877, and −0.967, corresponding to WFB, WPB20, WPB30, WPB40, and WPB50. This was due to the hardness and crystallinity gradually increased during the staling process of bread and the crumb moisture decreased. Therefore, the hardness, moisture, and crystallinity are important indicators for characterizing the staling of composed bread.

It is worth noting that the correlation coefficient between the hardness and crumb moisture as well as crystallinity suddenly decreased when the potato flour addition was about 50%, with the correlation coefficient were −0.635 and 0.539 respectively. During the experiment, an interesting phenomenon was observed that the composite bread with a high addition of potato flour preferred not readily to slag when sliced after staling, although their hardness values were greater than that of wheat bread. In the composite bread, the addition of gelatinized starch delayed the recrystallization of the starch, it is reasonable to suppose that the gelatinized starch changed the structure of the gluten network. This was probably because the PF did not have gluten network and thus hindered the process of water diffusion and transfer, as well as the interaction of starch and gluten (Joshi et al., 2018), moreover, the water redistribution could affect the localized amylopectin recrystallization kinetics (Besbes, Jury, Monteau, & Bail, 2014). Mehran, Behzad, Mostafa, and Saman (2018) found that there was a significant positive correlation between the moisture and homogeneity of the bread crumb. Therefore, it was preliminarily speculated that hardness is not necessarily an accurate judgment indicator in the process of bread staling. The relevant mechanism needs further experimental proof.

## 4 CONCLUSIONS

This study evaluated the quality and staling properties of composite bread, which formulated with different levels (0%, 20%, 30%, 40%, and 50%) of potato flour. With the addition of PF, the specific volume of composite bread decreased and the crust color became lighter. According to sensory evaluation, the composite bread was comparable to wheat bread when addition level of PF at 20%, but decreased when the addition level increased to 30% or more. From the comprehensive analysis of the sensory evaluation and texture tests, a chewy mouthfeel, an elastic and none‐crumbly texture of composite bread were observed, which had higher hardness value than wheat bread, and could keep on both longer linear distance and higher linear force, indicating that such new parameters other than hardness should be introduced to coordinate with the texture quality of composite bread.

The results of setback value from pasting behavior of the flours, as well as water migration and XRD during bread storage, evidenced that the addition of potato flour has an efficiency of antistaling of the composite bread. These indicators of bread staling characteristics showed high correlation coefficients between each other. The crumb moisture was negatively correlated with crystallinity and hardness, while crystallinity was positively correlated with hardness.

## ETHICAL REVIEW

This study was approved by the Institutional Review Board of Northwest Agriculture and Forest University.

## INFORMED CONSENT

Written informed consent was obtained from all study participants.
